# Recurrent NEDD4L Variant in Periventricular Nodular Heterotopia, Polymicrogyria and Syndactyly

**DOI:** 10.3389/fgene.2020.00026

**Published:** 2020-02-05

**Authors:** Katrien Stouffs, Patrick Verloo, Stefanie Brock, Luc Régal, Diane Beysen, Berten Ceulemans, Anna C. Jansen, Marije E.C. Meuwissen

**Affiliations:** ^1^ Center for Medical Genetics, UZ Brussel, Brussels, Belgium; ^2^ Neurogenetics Research Group, Reproduction Genetics and Regenerative Medicine Research Cluster, Vrije Universiteit Brussel, Brussels, Belgium; ^3^ Department of Pediatric Neurology, Ghent University Hospital, Ghent, Belgium; ^4^ Department of Pathology, UZ Brussel, Brussels, Belgium; ^5^ Pediatric Neurology Unit, Department of Pediatrics, UZ Brussel, Brussels, Belgium; ^6^ Department of Pediatric Neurology, Antwerp University Hospital, Edegem, Belgium; ^7^ Center of Medical Genetics, Antwerp University Hospital, Edegem, Belgium; ^8^ Center of Medical Genetics, University of Antwerp, Edegem, Belgium

**Keywords:** NEDD4L, periventricular nodular heterotopia, polymicrogyria, syndactyly, seizures, malformation of cortical development

## Abstract

NEDD4L encodes an ubiquitin ligase which is expressed in the cortex and ventricular zone of the fetal brain. Missense variants in NEDD4L have been reported in nine patients with periventricular nodular heterotopia (PNH), polymicrogyria, cleft palate, and syndactyly. All reported variants are located in the HECT domain, causing deregulation of signaling pathways, including the AKT/mTOR pathway. Here we describe a first familial case with four affected members with a high degree of intra-familial phenotypic variability. Phenotypic features in the proband consisted of severe neurodevelopmental delay, refractory seizures, bilateral PNH, and perisylvian polymicrogyria. The other family members were less severely affected with mild developmental delay and isolated bilateral PNH. All family members had syndactyly. An unrelated patient presented with severe neurodevelopmental delay, seizures, and hypospadias, expanding the phenotypic spectrum. MRI revealed bilateral PNH and perisylvian polymicrogyria. All tested patients carry the recurrent variant c.623G > A, p.(Arg208Gln) in the WW domain of NEDD4L. The variant in the unrelated patient occurred *de novo*. This is the first report of a NEDD4L variant located in the WW domain which is probably involved in the recognition of substrates for ligation suggesting a loss of function variant.

## Introduction

The development of the human cerebral cortex is complex and requires precise regulation of neuronal proliferation, migration, and postmigrational development. Nodules of immature neurons in the germinal zone, referred to as periventricular nodular heterotopias, are likely caused by disruption of the neuroependyma, leading to defective radial glia and impaired neuronal migration (Ferland et al., 2009). More recently, abnormal morphology of neuronal progenitor cells and defective migration have been reported in organoids as underlying cause of periventricular nodular heterotopia (PNH) (Klaus et al., 2019). Polymicrogyria, an excessive number of small gyri, is thought to be caused by defects in the pia membrane and in postmigrational neuronal differentiation (Barkovich et al., 2012).

NEDD4L is a HECT E3 ubiquitin ligase with multiple substrates regulating, among others, neuronal functions (Ekberg et al., 2014; Yanpallewar et al., 2016; Todaro et al., 2018). NEDD4L is highly expressed in the cortical plate, the ventricular zone, and the ganglionic eminences in the fetal brain of mice (Broix et al., 2016).

Recently, NEDD4L missense variants were identified in nine patients with periventricular nodular heterotopia (PNH), polymicrogyria (PMG), and variable features comprising 2-3 syndactyly of the toes, cleft palate, and epilepsy (Broix et al., 2016; Kato et al., 2017; Elbracht et al., 2018). All variants affected the HECT domain causing deregulation of the AKT/mTOR and TGF-ß/Smad2/3 signaling pathways (Broix et al., 2016; Kato et al., 2017). Four patients carried a recurrent mutation at p.(Glu893Lys) (Broix et al., 2016; Kato et al., 2017) and two variants at p.679 but with different substitutions have been reported (Broix et al., 2016; Elbracht et al., 2018), suggesting mutational hotspots. While six pathogenic variants occurred *de novo*, parental mosaicism accounts for three reported cases (Broix et al., 2016; Elbracht et al., 2018).

We report the first familial occurrence of a novel NEDD4L variant affecting the WW domain. The same variant occurred *de novo* in an additional case with a similar phenotype, suggesting a second mutational hotspot.

## Methods

After obtaining written parental consent, Sanger sequencing of all coding regions and part of the introns of the NEDD4L gene (NM_001144967.2) was performed in two families of Caucasian origin. Additionally, written parental consent was obtained for the publication of clinical and molecular data. This study has been approved by the ethical committee of UZ Brussel. DNA samples from all patients and parents were derived from peripheral blood. DNA extraction was performed using standard procedures in the referring center. PCR and Sanger sequencing were performed using standard conditions. Primer sequences are available in **Supplementary Data**. For the index cases, all coding regions of the NEDD4L gene were tested. Parents and sibs have only been analyzed for the detected variant. The interpretation of sequencing data is performed with Sequence Pilot (JSI). Paternity analysis in family 1 has been performed and confirmed using Complete V2 (Devyser).

## Case Reports

### Case 1

**Figure 1:**
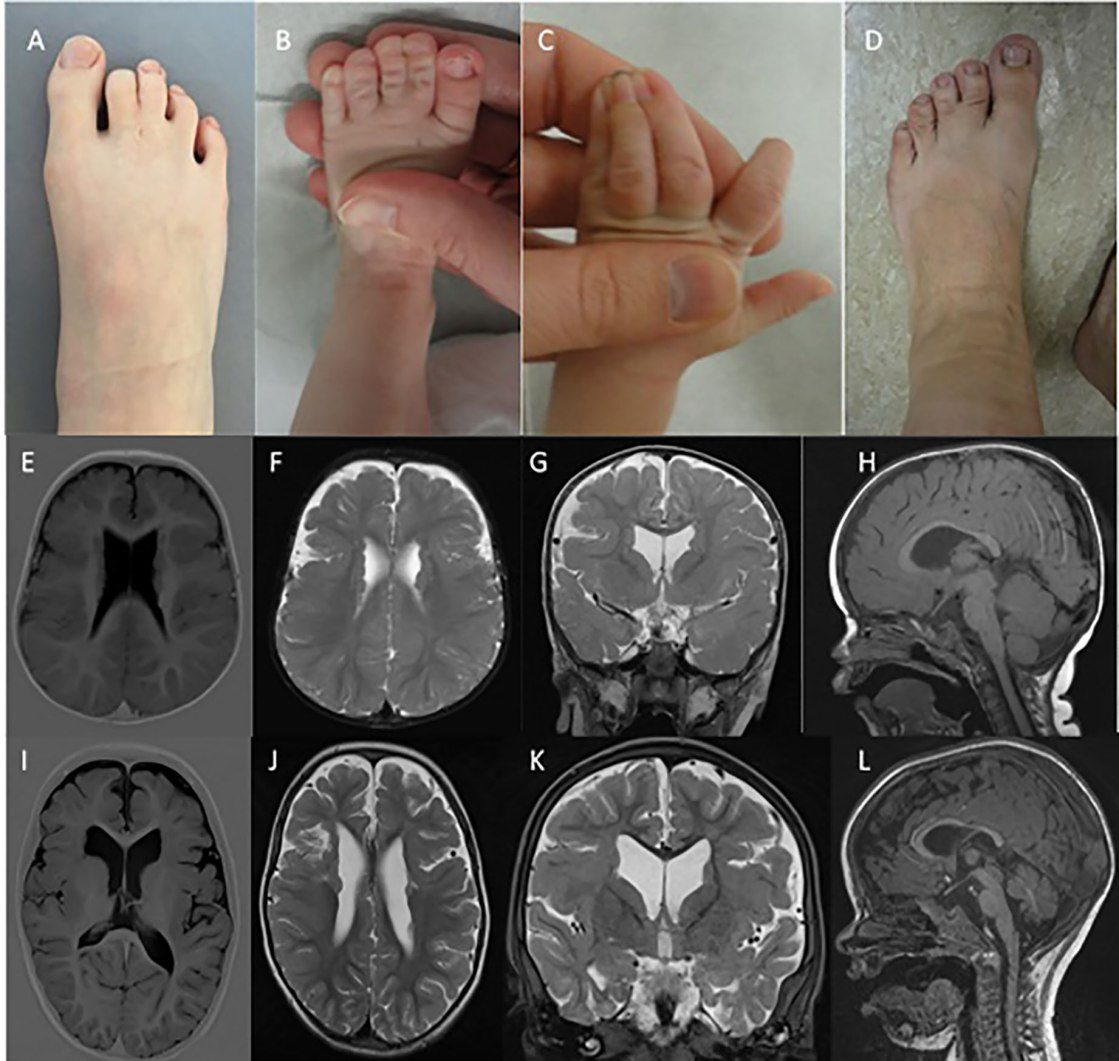
Syndactyly phenotypes and brain MRI. **(A D)** indicate the presence of variable syndactyly in case 1 **(A)**, case 2 **(B, C)**, and the mother of case 2 **(D)**. Brain MRIs of case 1 **(E–H)** and case 2 **(I–L)**. Mild ventricular dilatation, bilateral PNH, and bilateral PMG are demonstrated in both cases **(E-G, I-K)**. **(H**, **L)** demonstrate thinning of the corpus callosum with normal infratentorial findings.

The proband was born after a normal pregnancy and delivery with birth weight of 4,100 gram and height of 51 cm. The mother was treated for infantile seizures up to the age of 4 years, the father had a single febrile convulsion. Clinical examination revealed macrocephaly (SDS +2), prominent forehead, bilateral clinodactyly of the 5th fingers, partial cutaneous 2-3 syndactyly of the toes (**Figure 1A**), and penoscrotal hypospadias with bifid chordee. At the age of 20 months, mental age corresponded with 18 months and motor development with 14 months. He had febrile convulsions at the age of 20 months followed by focal seizures evolving to bilateral generalized convulsions for which he was treated for 2 years. At age 13 years, he is seizure free without medication. Brain MRI at age 14 months revealed bilateral PNH with overlying bilateral perisylvian PMG (**Figures 1E–H**). Brain MRI of both parents was normal.

### Case 2

The proband was born after normal pregnancy and delivery with birth weight of 2,754 gram. He had neonatal convulsions at age 14 days and status epilepticus at age 6 weeks. Seizures are very refractory to treatment, with frequent intensive care admissions for status epilepticus. Despite various antiepileptic treatment regimens, at the age of 4 he still suffers from clusters of focal and secondary generalized seizures. At the age of 23 months mental age corresponded with 9.5 months and motor development with 5–6 months. Speech is absent. He has generalized hypotonia, a high forehead with normal head circumference (-0.5 SD), cutaneous right-sided 3-4 syndactyly and a left-sided 3-4-5 syndactyly of the fingers, and bilateral 2-3 syndactyly of the toes (**Figures 1B, C**). Brain MRI revealed bilateral PNH with overlying perisylvian PMG (**Figures 1I–L**).

The proband's mother had infantile febrile convulsions. She needed special education, as did the proband's maternal half-sister and half-brother. The half-siblings had no seizures. Both mother and half-sister showed 2-3 toe syndactyly (**Figure 1D**). Brain MRI demonstrated PNH in all three.

## Results

For both patients, a gene panel analysis was performed for “malformations of cortical development” (v3 of the panel, see http://www.brightcore.be/mcd) but no pathogenic variants were found. However, at the time of the investigation, NEDD4L was not included in this gene panel. Based on the clinical phenotype combining PNH, PMG, and syndactyly it was decided to perform targeted Sanger sequencing of the NEDD4L gene in a second phase.

**Figure 2:**
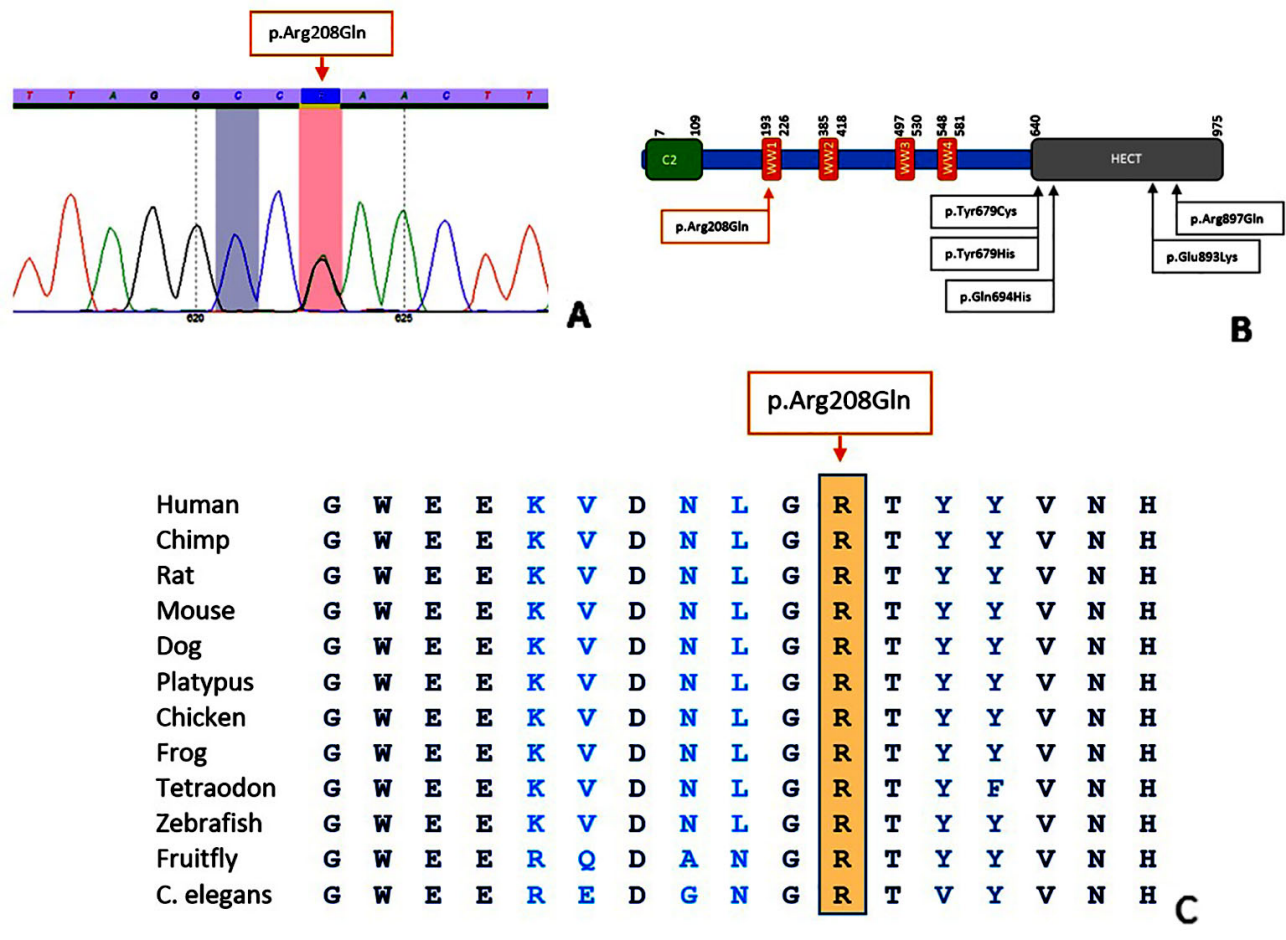
Characteristics of the p.(Arg208Gln) variant. Sequence analysis shows a G > A alteration at position 623 **(A)**. Location of the variant in the NEDD4L protein **(B)**; figure adapted from Kato et al., 2017. Conservation of the amino acid position up to C. elegans **(C)**; figure extracted from Alamut Visual (Version 2.11, Interactive Biosoftware).

In both families, a heterozygous c.623G > A, p.(Arg208Gln) variant in exon 9 of NEDD4L was identified. While the previously reported NEDD4L variants all affected the HECT domain, the variant in our patients is located in the WW domain (**Figure 2**). In Case 1, the variant was absent in both parents, indicating a *de novo* event. In Case 2, the variant was also identified in the affected mother and two half-siblings, segregating with the phenotype. The variant affects a highly conserved nucleotide (phyloP: 9.53) and amino acid and is absent in control databases (gnomAD, GoNl, dbSNP, ESP). Prediction programs PolyPhen2 and MutationTaster indicate a pathogenic effect (p value 1), while SIFT points to a polymorphism (score 0.05).

## Discussion

Our findings together with a review of the published cases highlight the co-occurrence of PNH and PMG as core features of the NEDD4L imaging phenotype. Patient 1 shows penoscrotal hypospadia as a novel finding. Patient 2 presents with an extensive syndactyly, including an unusual pattern of syndactyly of the fingers (**Figure 1C**). Our patients do not have cleft palate.

We describe the first familial occurrence, not associated with parental mosaicism, of a pathogenic variant in the NEDD4L gene with broad intra-familial phenotypic variability, varying from relatively well-functioning carriers with isolated PNH on brain MRI, to a severely affected case with profound developmental delay, refractory seizures, and PNH-PMG.

The NEDD4L variant in our patients affects the WW domain of the protein, while the previously reported variants were located in the HECT domain (Broix et al., 2016; Kato et al., 2017; Elbracht et al., 2018). WW domains can be found in different signaling proteins and contain two highly conserved tryptophan (W) residues. By forming monomeric protein binding modules *via* disulfide bridges, WW domains mediate protein-protein interactions. In NEDD4L, the WW domain is important for the recognition of substrates (Sudol et al., 1995; Salah et al., 2012; Spagnol et al., 2016). We hypothesize that conformational changes cause a loss of function of the WW domain leading to impaired recognition of substrates and ultimately to loss of protein function. Similar to the previously reported c.2677G > A, p.(Glu893Lys) variant in the NEDD4L HECT domain, the c.623G > A, p.(Arg208Gln) variant in the WW domain represents another potential mutational hotspot. Although the affected domain differs, no major phenotypic differences can be appreciated.

In conclusion, this report highlights the importance of detailed review of the cerebral cortex in all patients with PNH. The combination of PNH and PMG should prompt for careful evaluation for syndactyly, clefts, and/or hypospadias since these findings are highly suggestive of NEDD4L-involvement.

## Data Availability Statement

The variant detected in this study can be found in the LOVD database (#0000631993) (https://databases.lovd.nl/shared/variants/0000631993).

## Author Contributions

KS: drafting the manuscript, data analysis and interpretation, acquisition of data. PV: revising the manuscript, acquisition of data. SB: revising the manuscript, data analysis and interpretation. LR: revising the manuscript, acquisition of data. DB: revising the manuscript, acquisition of data. BC: revising the manuscript, acquisition of data. AJ: drafting and revising the manuscript, study concept, interpretation of data, study supervision. MM: drafting and revising the manuscript, study concept, interpretation of data, data acquisition, study supervision.

## Funding

AJ and KS received funding from the Scientific Fund Willy Gepts UZ Brussel.

## Conflict of Interest

The authors declare that the research was conducted in the absence of any commercial or financial relationships that could be construed as a potential conflict of interest.
