# Supplementary material for: Recurrent NEDD4L Variant in Periventricular Nodular Heterotopia, Polymicrogyria and Syndactyly
Source: Front Genet. 2020 Feb 5;11:26. doi: 10.3389/fgene.2020.00026 (PMC7013364; doi:10.3389/fgene.2020.00026)
Supplement: Supplementary file 1 [file Table_1.docx]

| NEDD4L- exon 1F | tgtaaaacgacggccagtCAGAGGAGGCTCGGAGGG |
| --- | --- |
| NEDD4L- exon 1R | caggaaacagctatgaccGGCGCGGGATAGGAACTC |
| NEDD4L- exon 2F | tgtaaaacgacggccagtCGAATTGATTTGTTCTGTATCCCAA |
| NEDD4L- exon 2R | caggaaacagctagtaccACTGTTGTGATGGTAAGTCCAGA |
| NEDD4L- exon 3F | tgtaaaacgacggccagtGTGTTGGGTCACATGAACAATGG |
| NEDD4L- exon 3R | caggaaacagctatgaccACTGTGTAAGTAGTCCATCCTTGAC |
| NEDD4L- exon 4F | tgtaaaacgacggccagtCCAGTTTGGTTTGTACGCTAGTC |
| NEDD4L- exon 4R | caggaaacagctatgaccTTGGGACACATTAGGAGGCTA |
| NEDD4L- exon 5F | tgtaaaacgacggccagtGGCGCATTGTAAAGCAGTATG |
| NEDD4L- exon 5R | caggaaacagctatgaccGAACATCAAACTCGTAGTGGGAC |
| NEDD4L- exon 6F | tgtaaaacgacggccagtGTGGTTACTCGTGTCTCCTCG |
| NEDD4L- exon 6R | caggaaacagctatgaccTACAGCCAGCTTCTCCTCCA |
| NEDD4L- exon 7F | tgtaaaacgacggccagtAAGAAACACATTCAGCGGTGC |
| NEDD4L- exon 7R | caggaaacagctatgaccCCCACACCACAGCAAGCA |
| NEDD4L- exon 8F | tgtaaaacgacggccagtGCTGAAGACGATTTTAAGTGTCC |
| NEDD4L- exon 8R | caggaaacagctatgaccTGCAAGTCCATAGCAATAATAGGA |
| NEDD4L- exon 9F | tgtaaaacgacggccagtCTAGAGCCAGAGAGCCCCA |
| NEDD4L- exon 9R | caggaaacagctatgaccTCTCGTACCACACCACCATC |
| NEDD4L- exon 10F | tgtaaaacgacggccagtCTCCGTGAGCATTGAACTGTC |
| NEDD4L- exon 10R | caggaaacagctatgaccATACGGCTTTTATAGGCACTTTCA |
| NEDD4L- exon 11F | tgtaaaacgacggccagtTCACCGGGGCTATTGTTGTTA |
| NEDD4L- exon 11R | caggaaacagctatgaccGATTCTCACCTGCTTGGGCTA |
| NEDD4L- exon 12F | tgtaaaacgacggccagtGAAGAGCGGTGAATATGGTTGAG |
| NEDD4L- exon 12R | caggaaacagctatgaccGAGATGGGTGGCAGTAACAATTC |
| NEDD4L- exon 13F | tgtaaaacgacggccagtGCATAGGGGTCACGCTTCC |
| NEDD4L- exon 13R | caggaaacagctagtaccTCTGTAGCACAAAGGAGCTAATC |
| NEDD4L- exon 14F | tgtaaacgacggccagtGACTCTGCAACTGTATGGGGTTC |
| NEDD4L- exon 14R | caggaaacagctatgaccCCAGGAAAGGAGTTCGGTCATGTA |
| NEDD4L- exon 15F | tgtaaaacgacggccagtGGGTTCGCGCTCCTAATCAC |
| NEDD4L- exon 15R | caggaaacagctatgaccCCACGGCCTTAGCCAAATGA |
| NEDD4L- exon 16F | tgtaaaacgacggccagtCTTGTCCATCTCCAAAGAGAAGCC |
| NEDD4L- exon 16R | caggaaacagctatgaccTCCAATTCCTCTGCAAGAACTACA |
| NEDD4L- exon 17F | tgtaaaacgacggccagtAGCCTGGTTGCCTTGAAACA |
| NEDD4L- exon 17R | caggaaacagctatgaccGAGATCCACAAAGCCTGGAGA |
| NEDD4L- exon 18F | tgtaaaacgacggccagtGCGTGTTGCTAGACATGGTCA |
| NEDD4L- exon 18R | caggaaacagctagtaccACCGTATGAAGGCACTGATTCTC |
| NEDD4L- exon 19F | tgtaaaacgacggccagtTAGAACACAGGGTGGGGGAC |
| NEDD4L- exon 19R | caggaaacagctatgaccATCTGAAAAGGCACATGCAGC |
| NEDD4L- exon 20F | tgtaaaacgacggccagtGTGTAGCGTACCCCAAATGGTT |
| NEDD4L- exon 20R | caggaaacagctatgaccACCAGGAGGATCAAAGTGCAGA |
| NEDD4L- exon 21F | tgtaaaacgacggccagtCTGTCTGCAAGGGAAAACCC |
| NEDD4L- exon 21R | caggaaacagctatgaccGCTGTGGGGTGAGGAGAGAA |
| NEDD4L- exon 22F | tgtaaaacgacggccagtTTTAGAGGGCACATGCGTCAG |
| NEDD4L- exon 22R | caggaaacagctatgaccTAAGCTACCAGAGAAAAGGACATC |
| NEDD4L- exon 23F | tgtaaaacgacggccagtGCTCATCTGTCACCTCCCAG |
| NEDD4L- exon 23R | caggaaacagctatgaccTCACATGGAATGCAACACCC |
| NEDD4L- exon 24F | tgtaaaacgacggccagtTCCAGAGTAGGTGCCTTCCA |
| NEDD4L- exon 24R | caggaaacagctatgaccGCTGGTTGTTTCTGATGGTCC |
| NEDD4L- exon 25F | tgtaaaacgacggccagtTTCCTGATAAATGAATAGCAGAGGA |
| NEDD4L- exon 25R | caggaaacagctatgaccACTGACCAATAAACTCATGCCCA |
| NEDD4L- exon 26F | tgtaaaacgacggccagtGGGTGTTCCAGGGAGTCTTG |
| NEDD4L- exon 26R | caggaaacagctagtaccATCAGCTCCACCACACTAGC |
| NEDD4L- exon 27F | tgtaaaacgacggccagtCACTGACATAGGAATCATCAGGAG |
| NEDD4L- exon 27R | caggaaacagctatgaccCATGTAATAGAACCACATTGGCA |
| NEDD4L- exon 28F | tgtaaaacgacggccagtACTGCTGCCTCTGTGATCTG |
| NEDD4L- exon 28R | caggaaacagctagtaccCAGTTTGCCTCTTCCCTCCC |
| NEDD4L- exon 29F | tgtaaaacgacggccagtTTAGAGAGGCCAGCTAGTATTGTG |
| NEDD4L- exon 29R | caggaaacagctatgaccATCCCTCGAAGTAGAAGCACCA |
| NEDD4L- exon 30F | tgtaaaacgacggccagtGAGAGTGTCTTGGGCCTCAC |
| NEDD4L- exon 30R | caggaaacagctatgaccTGCTGAACAGTGAAAGCTCC |
| NEDD4L- exon 31F | tgtaaaacgacggccagtTCTTACTCAAACCTCATGCCCTA |
| NEDD4L- exon 31R | caggaaacagctatgaccGAACTTGGACGAGTGCGTGG |
